# Development of a high sensitivity RT-PCR assay for detection of SARS-CoV-2 in individual and pooled nasopharyngeal samples

**DOI:** 10.1038/s41598-022-09254-1

**Published:** 2022-03-30

**Authors:** Harindi Jayakody, Daniel Rowland, Clint Pereira, Rachel Blackwell, Tomasz Lasota, Mark Laverick, Laurence Tisi, Hannah S. Leese, Alistair D. S. Walsham

**Affiliations:** 1 Erba Molecular, Ely, Cambridgeshire UK; 2 Materials for Health Lab, Department of Chemical Engineering, University of Bath, Bath, UK

**Keywords:** Molecular biology, Microbiology

## Abstract

The COVID-19 pandemic requires sensitive detection of the SARS-CoV-2 virus from samples to ensure accurate detection of infected patients, an essential component of effective national track and trace programs. Due to the scaling challenges of large sample numbers, sample pooling is an attractive solution to reduce both extraction and amplification reagent costs, if high sensitivity can be maintained. We demonstrate that the Erba Molecular ErbaMDx SARS-CoV-2 RT-PCR Kit (EM kit) delivers high sensitivity, achieving analytical detection of 5 copies/reaction SARS-CoV-2 genomic RNA, and 200 copies/mL SARS-CoV-2 inactivated virus spiked into nasopharyngeal swab (NP) samples and extracted through workflow. Furthermore, the EM Kit demonstrates high sensitivity in both pooled (1 in 5) and non-pooled NP samples when compared to an FDA Emergency Use Authorization approved assay, following published FDA guidelines. These findings demonstrate that the EM Kit is suitable for sample pooling, with minimal impact on assay performance. As the COVID-19 pandemic progresses, high sensitivity assays such as the EM Kit will have an important role in ensuring high throughput and sensitive testing using pooled samples can be maintained, delivering the most cost-effective sample extraction and amplification option for national test and trace programs.

## Introduction

COVID-19, an infectious disease caused by the severe acute respiratory syndrome coronavirus 2 (SARS-CoV-2) virus, was first reported in Wuhan, China in December 2019^1^. In March 2020, the WHO declared the COVID-19 outbreak a pandemic. As of November 2021, COVID-19 has accounted for over 250 million cases and over 5 million deaths globally^2^.

Transmission of SARS-CoV-2 is predominantly driven through contact with respiratory droplets which are released when a person coughs or sneezes, with aerosol and fomite contact being the primary infection routes^3^. Thus, diagnosis of SARS-CoV-2 infection has focussed on testing upper respiratory tract specimens including nasopharyngeal (NP) & oropharyngeal (OP) swabs, nasal mid-turbinate specimens, saliva, nasopharyngeal/nasal washes or aspirates and lower respiratory tract specimens^4^. Among the diagnostic platforms available for the testing of COVID-19, reverse transcription polymerase chain reaction (RT-PCR) has been presented as the most sensitive detection method for the diagnosis of SARS-CoV-2 infection^5^. NP swabs are considered the gold standard sample type for SARS-CoV-2 diagnostic testing^6^, despite concerns being raised about the accuracy of NP swab collection when self-collected (due to discomfort) and the potential risk of transmission of SARS-CoV-2 among health care professionals with assisted collection^6,7^.

As increased demand for mass testing has put strain on available resources, pooling has been suggested as a viable approach to enable large-scale testing of samples in environments where prevalence is low^8^. Pooling also facilitates high-throughput testing that is cost-effective, rapid and efficient through the process of combining multiple samples and performing a single extraction and PCR test on the combined sample^9–11^.

Pooling can be performed at different stages of the testing process. The US Food and Drug Administration (FDA) has identified two main approaches including swab pooling and sample/media pooling^8^. Swab pooling requires two samples to be collected from each patient if the need for re-testing to identify positive cases arises. Sample/media pooling on the other hand only uses a small volume of each sample, therefore multiple samples from individual patients are not required^12^. However, it is important to note that combining multiple samples in sample media pooling results in the dilution of the virus, increasing the likelihood of false negatives. Recognising the benefits of pooling in low prevalence settings (5–6%), the FDA has put forward guidelines to encourage manufacturers to validate molecular tests for pooling samples 1 in 5^8^. Therefore, pooling is most effective in low prevalence settings with the use of a validated highly sensitive assay which can mitigate false negative results^13^. The FDA has claimed that an individual test must achieve a positive percent agreement (PPA) of 85% or greater for pooled and non-pooled samples between itself and a comparator FDA Emergency Use Authorization (EUA) molecular assay, in order to receive FDA EUA approval as a pooling test^8^.

This study evaluates the performance of the Erba Molecular ErbaMDx SARS-CoV-2 RT-PCR Kit (EM kit), an assay which targets the Nucleocapsid (N1) and RNA dependent RNA polymerase (RdRp) genes of the SARS-CoV-2 viral genome, and the RNaseP gene of human genomic DNA (gDNA). The EM kit achieved 100% PPA in comparison to an FDA EUA molecular test using a 1 in 5 sample pooling strategy. This result demonstrates that the EM kit is a sensitive assay that can be used to detect SARS-CoV-2 positive samples with low viral loads in pooled samples.

## Results

### Analytical LoD of EM SARS-CoV-2 RT-PCR kit

Analytical Limit of Detection (LoD) was investigated to identify the minimum number of SARS-CoV-2 genomic RNA (gRNA) copies/reaction which demonstrated ≥ 95% detection using the ABI 7500 Real Time PCR machine. Tentative analytical LoD was estimated using a two-fold dilution series of SARS-CoV-2 gRNA (Supplementary information, Table S1) suggesting an LoD of 5 gRNA copies/reaction and between 20 and 40 gRNA copies/reaction for N1 and RdRp respectively, with a background of 1000 copies/reaction of human gDNA. The human gDNA concentration was chosen to provide a relevant background DNA level. However, SARS-CoV-2 gRNA may be present in samples with higher human gDNA concentration, which was not expected to impact overall assay performance. An analytical sensitivity of ≥ 95% detection was achieved for N1 at 5 gRNA copies/reaction and RdRp at 30 gRNA copies/reaction (Table 1). As detection of a single gene target is sufficient for a positive call, this demonstrated that the EM kit has an overall analytical LoD of 5 gRNA copies/reaction. A repeat of the LoD study using the BioRad CFX96 Opus PCR machine gave equivalent results (Table 1).

**Table 1:** LoD confirmation of analytical sensitivity of EM RT-PCR assay.

PCR instrument	gRNA/gDNA spike (copies/reaction)	Target	Avg Ct	S.D	Positive	Negative	Call rate (%)
ABI7500	5	N1	35.2	1.0	95	1	99
		RdRp	38.3	0.7	53	43	55.2
	1000	RNaseP	29.2	0.6	96	0	100
	30	N1	32.2	0.4	96	0	100
		RdRp	36.3	0.6	96	0	100
	1000	RNaseP	29.3	0.6	96	0	100
CFX96	5	N1	35.7	0.9	40	0	100
		RdRp	38.5	0.9	26	14	65
	1000	RNaseP	29.7	0.7	40	0	100
	30	N1	32.8	0.4	40	0	100
		RdRp	35.7	0.5	40	0	100
	1000	RNaseP	29.6	0.4	40	0	100

Each copy level was comprised of either 96 replicates (ABI7500) or 40 replicates (CFX96) at either 5 copies SARS-CoV-2 gRNA/reaction or 30 copies SARS-CoV-2 gRNA/reaction. Each reaction also contained 1000 copies of human gDNA (RNaseP) as an inhibitor control.

### Workflow LoD with NP and OP samples

Chosen extraction workflows were either manual (Qiagen QIAamp™ Viral RNA Mini Kit) or automated using KingFisher™ Duo Prime with manual addition of buffers to a 96 deep well plate (ThermoFisher MagMAX™ Viral/Pathogen Nucleic Acid Isolation Kit).

Workflow LoD was estimated for the Qiagen workflow using universal transport medium (UTM) spiked with 1000 copies human gDNA and a twofold dilution series of inactivated SARS-CoV-2 viral particles, suggesting an LoD between 150 copies/mL and 300 copies/mL for the N1 and RdRp targets (Supplementary information, Table S2). Qiagen workflow LoD was further assessed using the UTM model with inactivated SARS-CoV-2 viral particles, demonstrating an estimated LoD of 200 copies/mL for N1 and > 300 copies for RdRp (Supplementary information, Table S3). To confirm LoD, OP swab samples were spiked with 200, 400, and 450 copies of inactivated SARS-CoV-2 and extracted with the Qiagen workflow, with a LoD of 200 copies/mL for N1 and 450 copies/mL for RdRp achieved (Table 2). Due to OP swab performance, NP swab LoD with the Qiagen workflow was assessed using the same viral spike, 200 copies/mL for N1 and 450 copies/mL for RdRp, with ≥ 95% detection achieved for each target level (Table 2).

**Table 2:** LoD confirmation of workflow sensitivity for N1 and RdRp with Qiagen QIAamp™ Viral RNA mini kit (OP and NP swabs) and ThermoFisher MagMAX™ Viral/Pathogen Nucleic Acid Isolation Kit (NP swabs).

Extraction workflow	Viral spike (sample type)	Target gene	Avg Ct	SD	Positive	Negative	Call rate (%)
Qiagen	200 copies/ml (OP swab)	N1	35.2	1.0	20	0	100.0
		RdRp	37.8	0.9	19	1	95.0
		RNase P	29.2	2.5	20	0	100.0
	400 copies/ml (OP swab)	N1	33.5	1.4	19	1	95.0
		RdRp	37.4	0.8	17	3	85.0
		RNase P	28.1	1.6	20	0	100.0
	450 copies/ml (OP swab)	N1	33.2	0.5	20	0	100.0
		RdRp	36.7	0.7	19	1	95.0
		RNase P	30.5	2.5	20	0	100.0
	200 copies/mL (NP swab)	N1	33.7	1	20	0	100
		RdRp	37.5	0.8	18	2	90
		RNaseP	24.2	0.5	20	0	100
	450 copies/mL (NP swab)	N1	33.2	0.6	20	0	100
		RdRp	36.3	0.5	20	0	100
		RNaseP	24.9	0.4	20	0	100
ThermoFisher	200 copies/mL (NP swab)	N1	32.6	0.5	20	0	100
		RdRp	36.1	0.6	20	0	100
		RNaseP	24.8	0.4	20	0	100

Workflow sensitivity LoD was determined as ≥ 95% detection (19/20) of N1 or RdRp target with Ct values < 40 with eluates from each extraction workflow.

Workflow LoD was estimated for the ThermoFisher workflow using a twofold dilution series of inactivated SARS-CoV-2 virus spiked into pooled NP swab material, suggesting an LoD between 150 and 300 cps/mL for both the N1 and RdRp targets (Supplementary information, Table S4).” As we had previously demonstrated similar detection with the Qiagen workflow, 200 copies/mL and 450 copies/mL were initially selected to determine Thermofisher workflow LoD. However, as 100% detection was observed at 200 copies/mL for both the N1 and RdRp targets, the 450 copies/mL input level was not investigated (Table 2).

### Clinical performance

Positive percentage agreement (PPA) and negative percentage agreement (NPA) were 100%, demonstrating complete concordance with an FDA EUA comparator assay (DiaCarta QuantiVirus™ Real-time PCR coronavirus (SARS-CoV-2) detection assay) (Table 3).

**Table 3:** Clinical NP/OP swab evaluation of EM Kit compared to DiaCarta QuantiVirus™ Real-time PCR coronavirus (SARS-CoV-2) detection kit.

NP sample status	N	SARS-CoV-2		Sensitivity (95% CI)	Specificity (95% CI)	PPA (95% CI)	NPA (95% CI)
		Detected	Not detected				
Positive	39	39	0	100% (0.91–1.00)	100% (0.97–1.00)	100% (0.91–1.00)	100% (0.97–1.00)
Negative	110	0	110				

SARS-CoV-2 RNA was extracted from swab samples using the Qiagen QIAamp™ Viral RNA mini kit with eluates split and frozen at − 80 °C. Aliquots were then tested with both amplification kits.

### NP sample pooling validation

**Figure 1:**
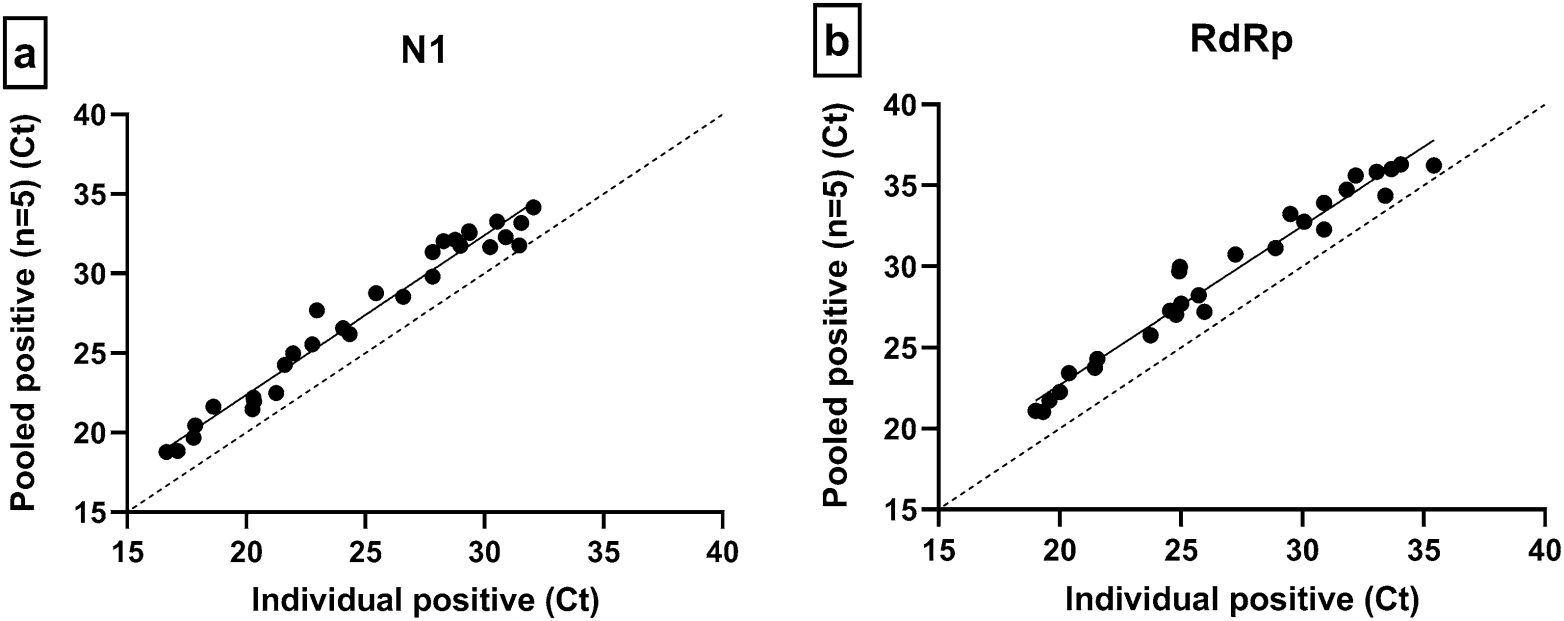
Regression curve analysis of individual positive Ct vs pooled positive Ct with EM Kit. Comparison of individual sample Ct (*x*) versus pooled sample Ct (*y*) for 30 SARS-CoV-2 positive NP swab samples. (**a**) N1 Ct comparison: + 2.4 Ct, 95% CI = 0.48–4.16, R^2^ = 0.97. (**b**) RdRp Ct comparison: + 2.6 Ct, 95% CI = 1.05–5.18, R^2^ = 0.96.

The EM kit demonstrated 100% PPA and NPA for detection of SARS-CoV-2 positive NP samples in both individual and pooled conditions compared to the DiaCarta QuantiVirus™ Real-time PCR coronavirus (SARS-CoV-2) detection assay (Table 4). Regression curve analysis demonstrated a high Ct correlation between individual and pooled samples, with an increase of 2.4 cycle threshold (Ct) for N1 and 2.6 Ct for RdRp (Fig. 1).

**Table 4:** Clinical NP sample pooling for SARS-CoV-2 detection with EM Kit.

NP sample status	N	SARS-CoV-2		Sensitivity (95% CI)	Specificity (95% CI)	PPA (95% CI)	NPA (95% CI)
		Detected	Not detected				
**Individual**							
Positive	30	30	0	100% (0.89–1.00)	100% (0.89–1.00)	100% (0.89–1.00)	100% (0.89–1.00)
Negative	30	0	30				
**Pooled**							
Positive	30	30	0	100% (0.89–1.00)	100% (0.89–1.00)	100% (0.89–1.00)	100% (0.89–1.00)
Negative	30	0	30				

Individual positive and negative samples were blinded, extracted using the Magmax™ Viral/Pathogen Nucleic Acid isolation kit and tested with both the EM Kit and the QuantiVirus™ Real-time PCR coronavirus (SARS-CoV-2) detection kit (individual). Each blinded sample was then pooled with 4 negative NP samples and tested with the EM Kit and compared to the individual QuantiVirus™ Real-time PCR coronavirus (SARS-CoV-2) detection kit (pooled).

## Discussion

Our findings demonstrate that the EM kit is a highly sensitive RT-PCR assay that can detect SARS-CoV-2 gRNA extracted from both non-pooled and 1 in 5 pooled NP swab samples. As SARS-CoV-2 viral loads have been demonstrated to vary substantially within patient samples, ranging from 9 copies/mL through to 1 × 10^10^ copies/mL, high assay sensitivity is essential to mitigate the risk of false negative results from positive samples with a low viral load^14,15^. The EM kit has demonstrated ≥ 95% detection of 5 copies gRNA/reaction analytically, and ≥ 95% detection of 200 copies/mL inactivated SARS-CoV-2 virus spiked into NP swab material. A 1 in 5 pooling strategy would increase the LoD of the assay to ~ 1000 copies/mL, and as typical SARS-CoV-2 viral loads lie above this threshold, the EM kit facilitates the accurate detection of positive samples in both non-pooled and pooled samples^16–20^.

The FDA guidelines for sample pooling recommends a PPA of 85% or above^8^. In this study, the use of a 5-sample pooling strategy with the EM kit in line with the FDA recommendation was investigated, and demonstrated 100% PPA and NPA with the comparator DiaCarta kit for both pooled and non-pooled samples. Given that the EM kit was 100% concordant with the comparator DiaCarta QuantiVirus™ SARS-CoV-2 test kit which has an LoD of 200 copies/mL and is considered as one of the most sensitive assays, this gives an indication that the sensitivity of the EM kit is comparable to the DiaCarta QuantiVirus™ test kit^16^.

Sample processing time and throughput are additional factors that determine the success of molecular diagnostic platforms in clinical settings i.e., high throughput platforms with longer turnaround times vs lower throughput platforms with rapid turnaround times. Current commercial molecular diagnostic platforms such as the Hologic Panther Fusion™ can process 120 samples within ~ 6.6 h whilst the PerkinElmer, ThermoFisher TaqPath and Roche cobas SARS-CoV-2 tests can process 94–96 samples within 2–3 h^21,22^. The EM kit is compatible with a semi-automated workflow (ThermoFisher MagMAX™ Viral/Pathogen Nucleic Acid Isolation Kit with processing using the KingFisher™ Duo Prime) which enables 12 samples to be extracted in < 30 min, and a RT-PCR protocol of < 90 min, demonstrating a turnaround time of approximately 2 h. If used with the Kingfisher™ Flex, the EM kit has the potential to process 96 samples within 2 h, which is comparable to fully automated kits, such as PerkinElmer^21^. Adopting the 5-sample pooling protocol will enable the EM kit to potentially process 480 samples within ~ 2 h. Whilst automation greatly improves sample processing throughput, it is essential that appropriate quality control measures are in place to reduce the risk of lab-based sample contamination.

Sample pooling allows more samples to be tested with the same extraction and amplification resources, which has the potential to deliver substantial cost-savings to national test and trace programs^23^. For instance, Barak et al. 2020 demonstrated that employing a sample pooling strategy spared 76% of RT-PCR kits during the pooling of 135,000 samples^24^. This is advantageous when viral prevalence within the population is low and when screening for asymptomatic individuals, as most samples are expected to be negative. The Dorfman pooling strategy describes the pooling efficiencies for different pool sizes based on positivity rates, where a pool size of 5 can increase the number of samples tested by 2.15–2.35 times for a positivity rate between 5–6%^8^. Further increasing the sample pool size may deliver additional cost-savings however, this must be balanced against the increased risk of false negative results^25^. Thus, adaptive pool size strategies based on viral prevalence are required with an understanding that low viral load positive samples may be missed with increasing pool sizes. An important consideration for pooling is that combining samples may mask those which are poor quality that would have been rejected if tested individually, increasing the risk of a false negative result. The inclusion of a sample adequacy control, such as RNaseP, when testing individual samples reduces the risk of false negative results, as the concentration of human gDNA within a swab sample has been demonstrated to be significantly correlated to pathogen positivity rates^26,27^. Thus, a negative sample with a high sample adequacy control Ct may be associated with poor sampling, rather than a genuine negative result and this may be identified and rejected if required by the amplification test criteria^26,27^. When pooling, this sample adequacy information is lost and will likely contribute to an increase in false negative test results, as poor-quality samples within a pool will not be identified for rejection and repeat testing. However, this consideration must be balanced against improved time and labour efficiencies when pooling is used^8,28^.

Whilst this study has investigated pooling of up to five separate NP samples, an alternative pooling approach is swab pooling, with the addition of swabs from multiple patients into a single transport medium vial. In this pooling approach, RT-PCR assay sensitivity is not impacted by volumetric sample dilution^8^. This may be advantageous for a single household, where a positive result would require the whole household to potentially self-isolate however, if used with disparate individuals, additional swabs would be required as it would be necessary to identify the positive patient(s) within the pool. Due to the well-documented discomfort associated with NP swab sampling, compliance may be reduced if multiple swabs are required^29^. Alternative sample types such as saliva could be employed for pooling and as saliva is easier to collect, this limits the risk of false negatives from incorrectly collected samples because of discomfort from NP swab sampling. It is possible that viral stability may be impacted in saliva without preservative, however, recent reports have suggested that viral RNA within non-supplemented saliva is stable for over one week at room temperature, supporting the use of saliva as a sample type^30,31^. Whilst consideration needs to be made with regards to variable viral loads within saliva compared to NP swabs, further development of pooling with saliva would be worthwhile due to ease of collection when compared to invasive NP swabs, the gold standard sample type, which can cause gagging and discomfort^32^. This is particularly important with the emergence of highly transmissible SARS-CoV-2 variants such as Omicron, as this could increase the risk of transmission between patient and healthcare workers if NP samples are acquired with assisted collection.

Assay sensitivity is an attribute of several factors including sample type, sample collection and RNA extraction method, time of sampling, molecular assay design the number of gene targets, presence of positive/negative/internal extraction controls, among others^5^. The EM kit acquires its sensitivity through several of these factors, including the use of NP swab samples which have been reported to be highly sensitive for SARS-CoV-2^33^. Selection of gene targets is a key design choice and thus the inclusion of multiple viral gene targets such as N1 and RdRP in the EM kit, reduces the likelihood of false negatives in the event of genomic mutation^34^. Use of the SARS-CoV-2 N1 gene as a molecular target may increase sensitivity, as previous studies have suggested that subgenomic copies of the N gene are present in patient samples which increases the amount of target available for detection and therefore, the EM kit sensitivity^35^. However, subgenomic N gene gRNA has also been shown to persist post-infection, thereby making diagnosis of individuals who have active infection versus those who are recovering more challenging^35^. It is important to note that distinguishing between samples from patients with active infection and patients who have recovered is a potential challenge for all molecular assays, as detection of pathogen nucleic acid does not necessarily indicate the presence of a viable pathogen, only that the pathogen has been present^36^. Nonetheless, given that the pandemic is a public health concern, identifying and isolating infected individuals to limit the spread of the virus is a priority and thus, highly sensitive RT-PCR tests remain an essential resource in the control of viral transmission^15^.

As the SARS-CoV-2 pandemic progresses and the proportion of the world’s vaccinated population increases, national testing strategies may be required to adapt from a high frequency of positive samples to a situation where samples are mostly negative. As this change occurs, sample pooling with detection using the EM kit offers an attractive solution to reduce the cost of sample testing whilst maintaining high sensitivity.

## Methods

### SARS-CoV-2 inactivated virus and genomic RNA

Quantified SARS-CoV-2 inactivated virus was obtained through BEI Resources, NIAID, NIH: SARS-Related Coronavirus 2, Isolate USA-WA1/2020, Gamma-Irradiated, NR-52287.

Quantified SARS-CoV-2 genomic RNA (gRNA) was obtained from Dr. Maria R. Capobianchi through BEI Resources, NIAID, NIH: Genomic RNA from SARS-Related Coronavirus 2, Isolate Italy-INMI1, NR-52498.

### Primer and probe selection

Primers were designed to amplify specific regions of the N1 and RdRp genes within conserved regions of the SARS-CoV-2 genome. Primers specific to human RNaseP were included as an internal control. In lieu of primer sequences, minimum information for the publication of real-time quantitative PCR experiments (MIQE) guidelines^37^ specify publication of the reference sequence, anchor nucleotide, and amplicon length: RdRp: NC_045512.2 (13163-16515), 2317, 100; N1: NC_045512.2 (28274-29533), 52, 72; RNaseP: NC_000010.11 (90871974-90908556), 68, 65^38^. Primers for the N1 and RdRp sequences were tested for inclusivity of all known SARS-CoV-2 genome sequences in silico on 24^th^ September 2020 using NCBI BLAST and were found to have 100% homology for at least one of the SARS-CoV-2 PCR targets. Additionally, in silico analysis of SARS-CoV-2 genome sequences from GISAID’s EpiCoV database on 20th December 2021 found the assay to be inclusive for all variants of concern listed by the CDC on 01st December 2021, including the variant of concern B.1.1.529, named Omicron. Primers for the N1, RdRp, and RNaseP sequences were tested for exclusivity against non-SARS-CoV-2 coronavirus and common respiratory pathogens on 25th September 2020 in silico using BLAST and no cross-reactivity was identified.

Due to issues with the SARS-CoV-2 template contaminating oligonucleotide suppliers^39^, all primers and probes were screened without SARS-CoV-2 target nucleic acid to ensure the EM kit was not contaminated with SARS-CoV-2 template.

### RT-PCR

The EM kit was prepared as described in the instructions for use (IFU)^40^. Briefly, dilution buffer was added to the lyophilised mastermix and vacuum-dried positive control. Resuspended mastermix was then dispensed into 48 wells/96 well plate. Eluate (10 µL), positive control, or dilution buffer (no template control) was added to the mastermix (final 20 µL reaction volume) and transferred to the RT-PCR machine (Applied Biosystems ABI 7500 RT-PCR machine or BioRad CFX96 Opus RT-PCR machine). Thermal cycling was performed for 10 min at 45 °C (RT step), 3 min at 95 °C (polymerase activation step), with 45 cycles 15 s at 95 °C (denaturation) and 30 s at 60 °C (annealing and polymerisation). Data were analysed for FAM (N1), JOE/HEX (RdRp), and ROX (Human RNaseP), with detection of either N or RdRp gene within 40 cycles (Ct) used for a positive call. For negative samples, detection of RNaseP within 40 Ct was required, otherwise the sample was determined as invalid. Positive (SARS-CoV-2 gRNA or supplied positive control) and negative (dilution buffer) amplification controls were performed with each RT-PCR amplification.

### Clinical samples

Deidentified clinical remnant NP swab samples, determined as positive or negative for SARS-CoV-2 by either CE-marked or FDA-cleared PCR assay, were purchased for assay development and validation from Boca Biolistics (Pompano Beach, Florida, United States) and BioIVT (Burgess Hill, West Sussex, United Kingdom).

Negative OP swab samples were donated by Erba Molecular staff during a 2 week donation period, where non-symptomatic staff were tested for SARS-CoV-2 at the start and end of the collection period using a 3rd party PCR testing service.

### Clinical performance

NP and OP swab samples were stored at − 80 °C on receipt and thawed when required. SARS-CoV-2 positive NP swab samples were handled under containment level 3 conditions until viral inactivation during RNA extraction by the addition of a manufacturer-supplied guanidine-based binding/lysis buffer.

To confirm the predicate status of clinical samples, all swab samples (60 assumed positive and 120 assumed negative from predicate tests) were re-tested with a 3rd party PCR kit (DiaCarta QuantiVirus™ Real-time PCR coronavirus (SARS-CoV-2) detection kit. Samples were discordant if negative against positive predicate status (14), inconclusive (16), or invalid (1). Eluates from 149 concordant samples were blinded and tested with the EM Kit.

### Viral RNA extraction

Negative NP or OP swab samples were either bulk pooled and spiked with a known concentration of inactivated SARS-CoV-2 virus before extraction (workflow limit of detection (LoD)) or extracted as supplied alongside positive NP swab samples (clinical performance, pooling study). Samples were handled inside a Class II microbial safety cabinet until viral inactivation by the addition of either Buffer AVL (Qiagen QIAamp™ Viral RNA Mini Kit) or Binding solution (ThermoFisher MagMAX™ Viral/Pathogen Nucleic Acid Isolation Kit).

Viral RNA was extracted from 200 µL samples using either QIAamp™ Viral RNA Mini Kit (manual) or MagMAX™ Viral/Pathogen Nucleic Acid Isolation Kit (automated using KingFisher™ Duo Prime). Samples were processed following manufacturer’s instructions with RNA eluted into 60 µL manufacturer-supplied elution buffer and either stored at − 80 °C or used immediately.

### Pooling study

The pooling study was designed following published guidance from the FDA^8^. The pooling study design specified by the FDA must include at least 25% of samples tested being low positive (within 2–4 Ct of the average LoD Ct) with 85% PPA of the non-pooled sample tested with a 3rd party comparator test. A total of 45 SARS-CoV-2 positive and 94 negative NP swab samples were extracted using the ThermoFisher MagMAX™ Viral/Pathogen Nucleic Acid Isolation Kit and screened using the EM Kit. Of the positive samples, 22 were chosen as medium to high positive samples with Ct ≤ 29. A further 8 samples were chosen as low positives with a Ct between Ct 29.1 and 31.1, which is within 2–4 Ct values of the average assay Ct at LoD (Ct 33.1). The 30 SARS-CoV-2 positive and 30 negative NP swab samples were randomised and blinded. Each sample was extracted using MagMAX™ Viral/Pathogen Nucleic Acid Isolation Kit and tested with both the EM Kit and the DiaCarta QuantiVirus™ Real-time PCR coronavirus (SARS-CoV-2) detection kit. Positive samples were then blinded, diluted 1 in 5 with NP swab material from 4 confirmed negative NP swab samples, extracted, and analysed with the EM Kit. After analysis, samples were unblinded and assay PPA and NPA values were determined.

### Data analysis

Real time RT-PCR data were processed using ABI 7500 software v2.0.6 (ABI 7500) or Bio-Rad CFX Maestro 2.0 v5.0.021 (Bio-Rad Opus 96). Ct values were interpreted as described in the manufacturer’ IFU for each molecular diagnostic assay. Data were analysed using Microsoft Excel 365 software (Microsoft, Redmond, WA). Figures were prepared using GraphPad Prism version 9.2.0 for Windows (GraphPad Software, San Diego, California USA), www.graphpad.com.

### PPA and NPA calculation

PPA and NPA values were calculated for the clinical specimens and pooling studies as follows:

$${\text{PPA}} = \left[ {{\text{true}}\,{\text{positives}}/\left( {{\text{true}}\,{\text{positives}} + {\text{false}}\,{\text{negatives}}} \right)} \right] \times 100$$

$${\text{NPA}} = \left[ {{\text{true}}\,{\text{negatives}}/\left( {{\text{false}}\,{\text{positives}} + {\text{true}}\,{\text{negatives}}} \right)} \right] \times 100$$

### Ethical declaration

All experimental protocols were performed in accordance with the ethical requirements of Erba Molecular. NP samples were collected under the suppliers own ethical approval process. OP samples were donated with informed consent by Erba Molecular staff. All methods were carried out in accordance with relevant national guidelines and regulations.

## Supplementary Information

Supplementary Information.

## Data Availability

All data generated or analysed during this study are included in this published article (and its Supplementary Information files).
