# Supplementary material for: Development of a high sensitivity RT-PCR assay for detection of SARS-CoV-2 in individual and pooled nasopharyngeal samples
Source: Sci Rep. 2022 Mar 30;12:5369. doi: 10.1038/s41598-022-09254-1 (PMC8965539; doi:10.1038/s41598-022-09254-1)
Supplement: Supplementary file 1 — Supplementary Information. [file 41598_2022_9254_MOESM1_ESM.docx]

**Development of a high sensitivity RT-PCR assay for detection of SARS-CoV-2 in individual and pooled nasopharyngeal samples**

Harindi Jayakody^1,2^, Daniel Rowland, Clint Pereira, Rachel Blackwell, Tomasz Lasota, Mark Laverick, Laurence Tisi^1^, Hannah S. Leese^2^, Alistair D. S. Walsham^1^*

^1^ Erba Molecular, Ely, Cambridgeshire, UK

^2^Materials for Health Lab, Department of Chemical Engineering, University of Bath, Bath, UK

*corresponding author

[a.walsham@erbamannheim.com](mailto:a.walsham@erbamannheim.com)

**Supplementary information**

**Table S1: Tentative analytical LoD of EM Kit using a two-fold dilution series.**

SARS-CoV-2 gRNA was diluted to the defined concentration (40, 20, 10, 5 copies/ reaction) and each dilution was tested 24 times using the ABI 7500 RT-PCR instrument.

|  | gRNA/ reaction | Target | Average Ct | SD | Positive | Negative | Call rate |
| --- | --- | --- | --- | --- | --- | --- | --- |
| ABI7500 | 40 cps | N1 | 31.0 | 0.3 | 24 | 0 | 100.0% |
|  |  | RdRp | 35.9 | 0.4 | 24 | 0 | 100.0% |
|  |  | RNaseP | 29.6 | 0.3 | 24 | 0 | 100.0% |
|  |  |  |  |  |  |  |  |
|  | 20 cps | N1 | 32.5 | 0.3 | 24 | 0 | 100.0% |
|  |  | RdRp | 37.6 | 0.7 | 22 | 2 | 91.7% |
|  |  | RNaseP | 29.6 | 0.3 | 24 | 0 | 100.0% |
|  |  |  |  |  |  |  |  |
|  | 10 cps | N1 | 34.1 | 0.5 | 24 | 0 | 100.0% |
|  |  | RdRp | 38.5 | 0.7 | 20 | 4 | 83.3% |
|  |  | RNaseP | 29.7 | 0.3 | 24 | 0 | 100.0% |
|  |  |  |  |  |  |  |  |
|  | 5 cps | N1 | 35.2 | 0.6 | 24 | 0 | 100.0% |
|  |  | RdRp | 38.9 | 0.4 | 7 | 17 | 29.2% |
|  |  | RNaseP | 29.8 | 0.5 | 24 | 0 | 100.0% |

**Table S2: Tentative Qiagen workflow LoD of EM Kit using a two-fold dilution series.**

UTM was spiked with known concentrations of inactivated SARS-CoV-2 virus and human genomic DNA and extracted with the Qiagen QIAamp™ Viral RNA Mini Kit. Eluates from each extraction were then tested with the EM Kit.

|  | Viral spike level | Target | Average Ct | SD | Positive | Negative | Call rate |
| --- | --- | --- | --- | --- | --- | --- | --- |
| ABI7500 | 600 copies/ ml | N1 | 33.0 | 0.4 | 8 | 0 | 100.0% |
|  |  | RdRp | 36.4 | 0.6 | 8 | 0 | 100.0% |
|  |  | RNaseP | 32.4 | 0.4 | 8 | 0 | 100.0% |
|  |  |  |  |  |  |  |  |
|  | 300 copies/ ml | N1 | 34.3 | 0.4 | 8 | 0 | 100.0% |
|  |  | RdRp | 38.1 | 0.7 | 8 | 0 | 100.0% |
|  |  | RNaseP | 32.8 | 0.3 | 8 | 0 | 100.0% |
|  |  |  |  |  |  |  |  |
|  | 150 copies/ ml | N1 | 36.1 | 1.1 | 7 | 1 | 87.5% |
|  |  | RdRp | 38.4 | 0.4 | 6 | 2 | 75.0% |
|  |  | RNaseP | 32.9 | 0.4 | 8 | 0 | 100.0% |
|  |  |  |  |  |  |  |  |
|  | 75 copies/ ml | N1 | 36.1 | 0.5 | 6 | 2 | 75.0% |
|  |  | RdRp | 39.2 | 0.8 | 3 | 5 | 37.5% |
|  |  | RNaseP | 32.9 | 0.3 | 8 | 0 | 100.0% |

**Table S3: Tentative Qiagen workflow LoD of EM Kit at 200 and 300 copies/ mL**

UTM was spiked with 200 cps/ mL or 300 cps/ mL inactivated SARS-CoV-2 virus and human genomic DNA and extracted with the Qiagen QIAamp™ Viral RNA Mini Kit. Eluates from each extraction were then tested with the EM Kit.

|  | Viral spike level | Target | Average Ct | SD | Positive | Negative | Call rate |
| --- | --- | --- | --- | --- | --- | --- | --- |
| ABI7500 | 200 copies/ mL | N1 | 34.4 | 34.4 | 31 | 1 | 96.9% |
|  |  | RdRp | 37.8 | 37.8 | 28 | 4 | 87.5% |
|  |  | RNaseP | 32.3 | 32.3 | 32 | 0 | 100.0% |
|  |  |  |  |  |  |  |  |
|  | 300 copies/ mL | N1 | 35.6 | 35.6 | 32 | 0 | 100.0% |
|  |  | RdRp | 38.0 | 38.0 | 29 | 3 | 90.6% |
|  |  | RNaseP | 33.4 | 33.4 | 32 | 0 | 100.0% |

**Table S4: Tentative ThermoFisher workflow LoD of EM Kit using a two-fold dilution series.**

SARS-CoV-2 negative NP samples were spiked with known concentrations of inactivated SARS-CoV-2 virus and extracted with ThermoFisher MagMAX™ Viral/Pathogen Nucleic Acid Isolation Kit. Eluates from each extraction were then tested with the EM Kit.

|  | Viral spike level | Target | Average Ct | SD | Positive | Negative | Call rate |
| --- | --- | --- | --- | --- | --- | --- | --- |
| ABI7500 | 600 copies/ ml | N1 | 30.0 | 0.6 | 8 | 0 | 100.0% |
|  |  | RdRp | 34.2 | 0.2 | 8 | 0 | 100.0% |
|  |  | RNaseP | 24.0 | 0.3 | 8 | 0 | 100.0% |
|  |  |  |  |  |  |  |  |
|  | 300 copies/ ml | N1 | 30.9 | 0.6 | 8 | 0 | 100.0% |
|  |  | RdRp | 35.0 | 0.4 | 8 | 0 | 100.0% |
|  |  | RNaseP | 23.5 | 0.6 | 8 | 0 | 100.0% |
|  |  |  |  |  |  |  |  |
|  | 150 copies/ ml | N1 | 32.1 | 0.9 | 7 | 1 | 87.5% |
|  |  | RdRp | 36.3 | 1.3 | 7 | 1 | 87.5% |
|  |  | RNaseP | 23.7 | 0.4 | 8 | 0 | 100.0% |
|  |  |  |  |  |  |  |  |
|  | 75 copies/ ml | N1 | 33.3 | 0.7 | 8 | 0 | 100.0% |
|  |  | RdRp | 36.4 | 0.5 | 8 | 0 | 100.0% |
|  |  | RNaseP | 23.9 | 0.4 | 8 | 0 | 100.0% |
